# LEON-BIS: multiple alignment evaluation of sequence neighbours using a Bayesian inference system

**DOI:** 10.1186/s12859-016-1146-y

**Published:** 2016-07-07

**Authors:** Renaud Vanhoutreve, Arnaud Kress, Baptiste Legrand, Hélène Gass, Olivier Poch, Julie D. Thompson

**Affiliations:** Department of Computer Science, ICube, UMR 7357, University of Strasbourg, CNRS, Fédération de médecine translationnelle de Strasbourg, Strasbourg, France

**Keywords:** Homology-based methods, Multiple sequence alignment, Sequence homology, Bayesian statistics

## Abstract

**Background:**

A standard procedure in many areas of bioinformatics is to use a multiple sequence alignment (MSA) as the basis for various types of homology-based inference. Applications include 3D structure modelling, protein functional annotation, prediction of molecular interactions, etc. These applications, however sophisticated, are generally highly sensitive to the alignment used, and neglecting non-homologous or uncertain regions in the alignment can lead to significant bias in the subsequent inferences.

**Results:**

Here, we present a new method, LEON-BIS, which uses a robust Bayesian framework to estimate the homologous relations between sequences in a protein multiple alignment. Sequences are clustered into sub-families and relations are predicted at different levels, including ‘core blocks’, ‘regions’ and full-length proteins. The accuracy and reliability of the predictions are demonstrated in large-scale comparisons using well annotated alignment databases, where the homologous sequence segments are detected with very high sensitivity and specificity.

**Conclusions:**

LEON-BIS uses robust Bayesian statistics to distinguish the portions of multiple sequence alignments that are conserved either across the whole family or within subfamilies. LEON-BIS should thus be useful for automatic, high-throughput genome annotations, 2D/3D structure predictions, protein-protein interaction predictions etc.

## Background

Multiple alignments of protein sequences are a fundamental tool in many areas of molecular biology, including phylogenetic studies, prediction of 2D/3D structure, or propagation of structural/functional information from annotated to non-annotated sequences. All of these applications rely on the identification of the conserved regions in the alignments, suggesting potential homologous relations between the sequences. However, downstream results may be highly sensitive to the alignment used, and ignoring errors or uncertainty in the alignment can lead to significant bias in the subsequent inferences [1]. For example, in evolutionary studies, it has been shown that the accuracy of phylogenetic trees inherently depends on the accuracy of the underlying sequence alignment [2]. Similarly, the CASP comparative modelling experiments [3] have regularly demonstrated that the quality of sequence alignments is a key factor in comparative modelling of protein 3D structures. Furthermore, many functional predictions are made on the basis of homology with remotely related sequences or structures. In this case, if functional predictions are to be made with confidence, information on the reliability of the alignment at the different sites is critical.

As a consequence, protein MSA is an exceptionally active field of research, and one of the latest developments has been a gradual shift away from the development of more accurate aligners towards the estimation of the reliable regions of an alignment. For example, numerous column scores have been defined that attempt to distinguish the positions that are conserved in all the sequences from the unreliable positions, e.g. [4, 5]. Typically, the unreliable positions are then filtered in the subsequent inference methods, or the methods can be used to identify interesting alignment blocks for phylogenetic studies, e.g. [6, 7]. In [8], the authors compared 8 state of the art methods of alignment trimming for phylogenetic studies, and used the annotated ‘core blocks’ from the BAliBASE benchmark [9] as the gold standard definition of reliable positions. However, it has been shown recently that the trees obtained from these filtered MSAs may be worse than those obtained from unfiltered MSAs in some cases [2].

An alternative approach involves the detection of conserved regions in the aligned sequences, for example using consensus sequences to define conserved alignment blocks [10,11]. More recently, DivA [12] used four divergence-based parameters and their outlier values to identify very divergent segments in MSAs. The method was used to identify badly annotated introns/exons in sets of orthologous proteins generated by a large-scale avian phylogenomics project. Another recent method, OD-seq [13], is designed to find outlier sequences by examining the average distance of each sequence to the rest and represents a useful, fast method for checking very large alignments containing thousands of sequences.

In general, these methods work well for multiple alignments of proteins that are homologous over their full lengths and allow the accurate detection of regions that are conserved in all members of a sequence family. However, large multi-domain proteins are becoming more and more prevalent in the sequence databases, with the arrival of numerous new genome sequences, in particular from eukaryotic organisms. In addition, badly predicted sequences mean that there are numerous fragments, spurious insertions/deletions and ‘incoherent’ segments in any set of sequences retrieved from the generalist databases [1]. In the face of these highly complex proteins, new methods are needed to detect local homology corresponding to structural/functional domains or motifs, and in particular those that explain the specificities of certain subfamilies. For example, for detection of binding on different substrates or cofactors and distinct binding affinities [14], or for residue-level genotype-phenotype correlation studies [15].

We previously developed LEON [16] to predict homologous regions in MSAs with respect to a user-defined reference or “query” sequence, and to identify non-homologous or outlier sequences. LEON incorporated sequence clustering [17] and calculated amino acid frequency profiles [18] in order to identify locally conserved motifs or ‘core blocks’ within the subfamilies. The conserved blocks for each subfamily were then chained together to form contiguous regions. In large-scale tests, where the conserved regions detected by LEON were compared to known structural or functional domains, the specificity of LEON was shown to be very high (>99 %), although at the expense of some loss of sensitivity (76 %) which meant that some divergent sequences were removed from the alignments even though they were actually related.

Here we introduce a new version of LEON, called LEON-BIS, that replaces the original amino acid frequency profiles by more robust Bayesian statistics based on BILD scores [19]. Bayesian methods provide a natural and principled way of combining prior information with data, within a solid decision theoretical framework. Past information about a parameter can be incorporated to form a prior distribution for future analysis. When new observations become available, the previous posterior distribution can be used as a prior. All inferences logically follow from Bayes’ theorem and they are robust to errors and missing data. When the sample size is large, Bayesian inference often provides results that are very similar to the results produced by frequentist methods. However, because Bayesian analyses do not assume large samples, smaller data sets can be analyzed without losing power but retaining precision. A Bayesian approach has been used previously to construct local pairwise alignments [20] or to perform a joint analysis of multiple sequence alignments and evolutionary trees [21], for example. We have also incorporated a similar Bayesian framework in the SIBIS method [22] to detect inconsistent sequence segments, often corresponding to badly predicted intron/exon structures in protein sequences.

The accuracy of the LEON-BIS method for the detection of conserved sequence segments is evaluated in a large-scale test, using more than 200 multiple sequence alignment from the latest version of the BAliBASE benchmark [23]. These alignments contain examples of many problems encountered in high-throughput projects, including complex multi-domain proteins, with locally conserved regions/core blocks, transmembrane proteins, fragments and badly predicted sequences, etc. In the final LEON-BIS alignment, the sequences that are predicted to be related to the user’s query sequence are ranked according to their similarity to the query sequence. Unrelated sequences containing no conserved regions are filtered from the alignment. More importantly, the conserved regions within the related sequences are delimited and can be thus used for reliable function annotation, fold classification, 2D/3D structure predictions, domain determination etc.

## Methods

### Training and test sets

To test the performance of the program, we used the most recent test set (Reference 10) in the BAliBASE benchmark suite [23], composed of 218 reference alignments and containing a total of 17,892 protein sequences. These reference alignments are designed to reflect some of the problems specific to aligning large sets of complex sequences. For example, many of the protein families have multi-domain architectures and their members often share only a single domain. In addition, the alignments have a high proportion of sequences with ‘discrepancies’ (unexpected or discordant extensions, insertions or deletions) that may correspond to naturally occurring variants or may be the result of artifacts, including proteins translated from partially sequenced genomes or ESTs, or badly predicted protein sequences. Taken together, this means that only a small proportion (18 %) of the conserved ‘core blocks’ in the alignments are present in most (>90 %) of the aligned sequences, while most of the blocks are only conserved within specific sub-families. These ‘rare’ segments or patterns are often characteristic of context-specific functions, e.g. substrate binding sites, protein-protein interactions or post-translational modification sites.

To create a suitable test set for the LEON-BIS evaluation experiments, we used the unaligned sequence sets corresponding to each reference alignment in BAliBASE Reference 10. The first sequence of each set was arbitrarily defined as the query sequence. For each set, we then added up to four sequences which were considered to be “unrelated” to the query sequence, by selecting sequences from the other reference alignments with two criteria: (i) the selected sequence shared less than 50 % percent residue identity with the query sequence and (ii) no shared domains were identified in the PFAM protein family database [24]. Finally, we aligned each of the reference sets containing both related and unrelated sequences using the MAFFT version 7 multiple alignment program [25].

### Algorithm overview

**Fig. 1:**
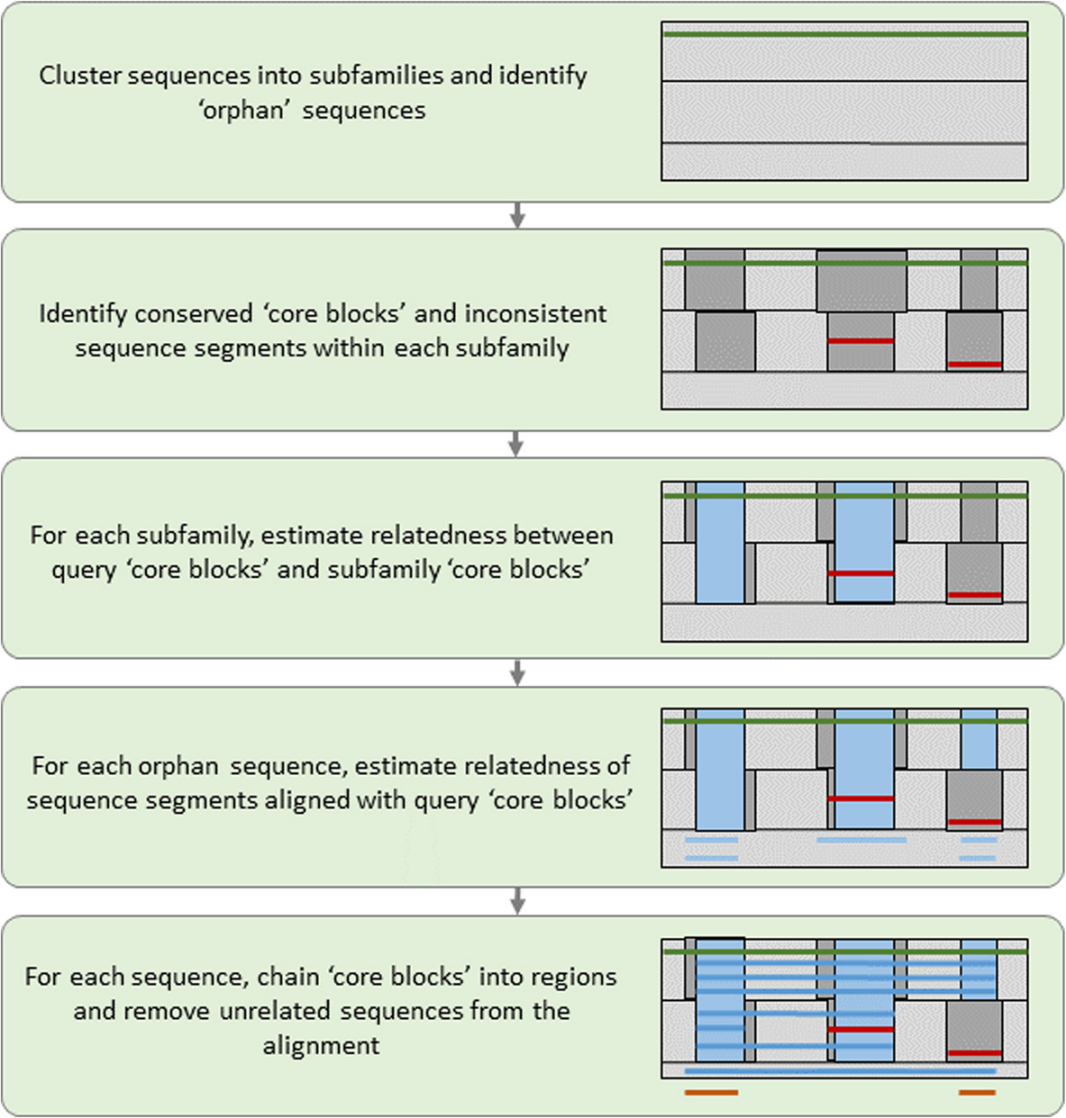
Schematic diagram of the steps involved in the LEON-BIS method

Given a multiple sequence alignment, LEON-BIS predicts, for each sequence in the alignment, the regions that are homologous to a specified query sequence. The algorithm uses a similar workflow to LEON [16] and consists of five major steps, as outlined in Fig. 1. In the first step, the protein sequences are clustered into subfamilies using the Secator program [17] and any highly divergent or 'orphan’ sequences in the alignment are identified and excluded from the subfamily clustering. In the second step, locally conserved segments or ‘core blocks’ are defined for each subfamily, based on Bayesian column scores and a sliding window analysis. At this stage, the SIBIS algorithm [22] is used to identify any ‘inconsistent’ sequence segments that may represent badly predicted sequences. In the third step, we estimate the relatedness of each core block in each subfamily with the core blocks in the query subfamily. In the fourth step, the identified orphan sequences are compared with the query subfamily, again using the SIBIS algorithm. The fifth and final step is the same as in the LEON program: conserved blocks that match the query sequence are chained together to form ‘regions’ and any sequences with no conserved regions are removed from the alignment.

### Identification of subfamily core blocks

For each subfamily within a multiple alignment, we use the approach described in [19] to calculate Bayesian Integral Log-odds (BILD) scores for each column in the subfamily. BILD scores represent the ratio of the probabilities of observing the amino acids in the alignment column under the assumption of relatedness, and under the assumption of non-relatedness. BILD scores are defined as:

$$ S\left(\underset{x}{\to}\right)= log\frac{Q\left(\underset{x}{\to}\right)}{P\left(\underset{x}{\to}\right)}={\displaystyle {\sum}_{k=1}^M log\frac{Prob\left({x}_k\Big|{\theta}_{k-1}\right)}{p_{x_k}}} $$

where the vector $\underset{x}{\to }$ of length M represents the data associated with the column and *θ*_*k*_ is the posterior distribution after observation of x_k_. They can be understood simply as the sum of log-odds scores for the individual letters observed in a column, with the “target frequency” for each letter x_k_ calculated based upon the prior distribution *θ*_0_ and the “previously observed” letters x_1_ through x_k-1_.

In our method, the prior probability distributions of the amino acids are described by Dirichlet distributions or Dirichlet mixtures. To our knowledge, the only Dirichlet mixture prior parameters for protein sequence alignments have been derived by the team who first proposed such mixtures [26] and these have been made available at compbio.soe.ucsc.edu/dirichlets/index.html. In these tests, we used a 20 component Dirichlet mixture (recode3.20comp), which was derived from analyses of large numbers of alignments of related protein sequences, and has a relative entropy of 0.61, roughly equivalent to that of the PAM-175 matrix.

A sliding window analysis of the BILD column scores for each subfamily is then performed. As BILD column scores are expressed as probabilities with values ranging between 0 and 1, we can define a threshold above which columns are considered to have significant scores. Here, core blocks are defined as segments with mean BILD score above 0.05.

### Estimation of relatedness between core blocks

Given two core blocks in different subfamilies that overlap in the multiple alignment, we estimate their relatedness using a Bayesian framework. Briefly, for two alignment columns, Altschul [19] defined substitution scores *R* for aligning two different columns of amino acids. Specifically, letting $\underset{xy}{\to }$ be the concatenation of the vectors $\underset{x}{\to }$ and $\underset{y}{\to }$, define:

$$ R\left(\underset{x}{\to },\underset{y}{\to}\right)=S\left(\underset{xy}{\to}\right)-S\left(\underset{x}{\to}\right)-S\left(\underset{y}{\to}\right)= log\frac{Q\left(\underset{xy}{\to}\right)}{Q\left(\underset{x}{\to}\right)Q\left(\underset{y}{\to}\right)} $$

where S is the BILD column score as defined above, and Q is the probability of observing the data under the assumption of relatedness.

We extend this to the case of aligning two core blocks by calculating the sum of the substitution scores for each of the aligned pairs of columns. The core blocks are assumed to be related if the sum of the column-column alignment scores is > 0.

### Estimation of relatedness between sequence segments and core blocks

In order to avoid including badly predicted or ‘inconsistent’ sequence segments in the predicted regions, the algorithm used in SIBIS [22] is implemented in LEON-BIS in order to calculate a score for the alignment of a single sequence segment to a core block. Briefly, the posterior distributions Θ_M_ after observing the alignment column x_1_ to x_M_ are used to calculate the probability of observing a new residue x_M+1_, under the assumption of relatedness. Then, the score for a segment of length N aligning to the core block (under the simplifying assumption that each position in the protein is generated independently) is equal to the product of the probabilities of aligning each residue to the corresponding column in the core block. In order to estimate the probability of observing a sequence segment under the assumption of unrelatedness, we calculate the score of a random sequence equal to the length of the core block with background amino acid frequencies equal to 1/L. Finally, sequence segments with a score less than that obtained by the random sequence are flagged as inconsistent sequences.

### Definition of regions and removal of unrelated sequences

Once the conserved core blocks are defined for all the sequences in the alignment, we chain these core blocks into larger ‘regions’, using the chain_blocks program implemented in the original LEON method. Then, the score for a conserved region is defined as the sum of the scores for the core blocks within the region. Sequences with no regions having a score higher than the cutoff value are removed from the alignment. For comparison purposes, the maximum distance between core blocks and the minimum length of a region are set to 40 and 21 respectively, the same as for the original LEON algorithm.

## Results

### Sequence-level homology analysis

To evaluate the accuracy of LEON-BIS for the detection of related and unrelated sequences, we constructed a large scale test set, based on the latest multiple alignments in the BAliBASE benchmark suite [23]. These alignments represent large complex protein families and include multi-domain proteins, transmembrane proteins, fragments, badly predicted sequences, etc. For the purpose of these experiments, we then added to each protein family a number of sequences known to be unrelated. Finally, the resulting sequence sets, containing both divergent sequences and unrelated sequences, were re-aligned using the MAFFT multiple alignment program [25]. Given a specific query sequence, LEON-BIS was used to predict the related and unrelated sequences in each alignment, and we estimated the accuracy of our approach in terms of sensitivity (=true positives/(true positives + false negatives)) and specificity (=true negatives/(true negatives + false positives)), shown in Table 1. We also compared the performance of our Bayesian-based method with two previously published algorithms, namely LEON [16] and OD-seq [13].

**Table 1:** Accuracy of three methods for the detection of related and unrelated sequences

	LEON related	LEON non-related	OD-seq related	OD-seq non-related	LEON-BIS related	LEON-BIS non-related
Related sequences	15,227	2304	17,298	513	15,552	1999
Non-related sequences	221	930	540	331	187	944
Total	15,448	3234	17,838	844	15,739	2943
Sensitivity	0.87		0.97		0.89	
Specificity	0.81		0.38		0.83	

LEON-BIS predicted slightly more related sequences (15,552 of all 18,682 sequences) compared to LEON (15,227), with higher sensitivity (0.89 versus 0.87), and higher specificity (0.83 versus 0.81). Thus, more sequences with hypothetical relationships are retained in the alignments, although some potential false positives are also included. However, it should be noted that the sequences in these test alignments were aligned using an automatic method, namely MAFFT, and so some of the sequences may be misaligned. Therefore, some of the false negative predictions are in fact true negatives, since the sequences do not contain any regions that are correctly aligned with the query sequence. OD-seq was less successful in these tests, but the authors themselves stated that the method was optimized for very large alignments and was not intended for small, very divergent families [13]. Consequently, the computation time required for the analysis of the 218 multiple alignments was significantly faster for OD-seq (27 s) compared to LEON or LEONII which required 1266 s and 2567 s respectively.

**Fig. 2:**
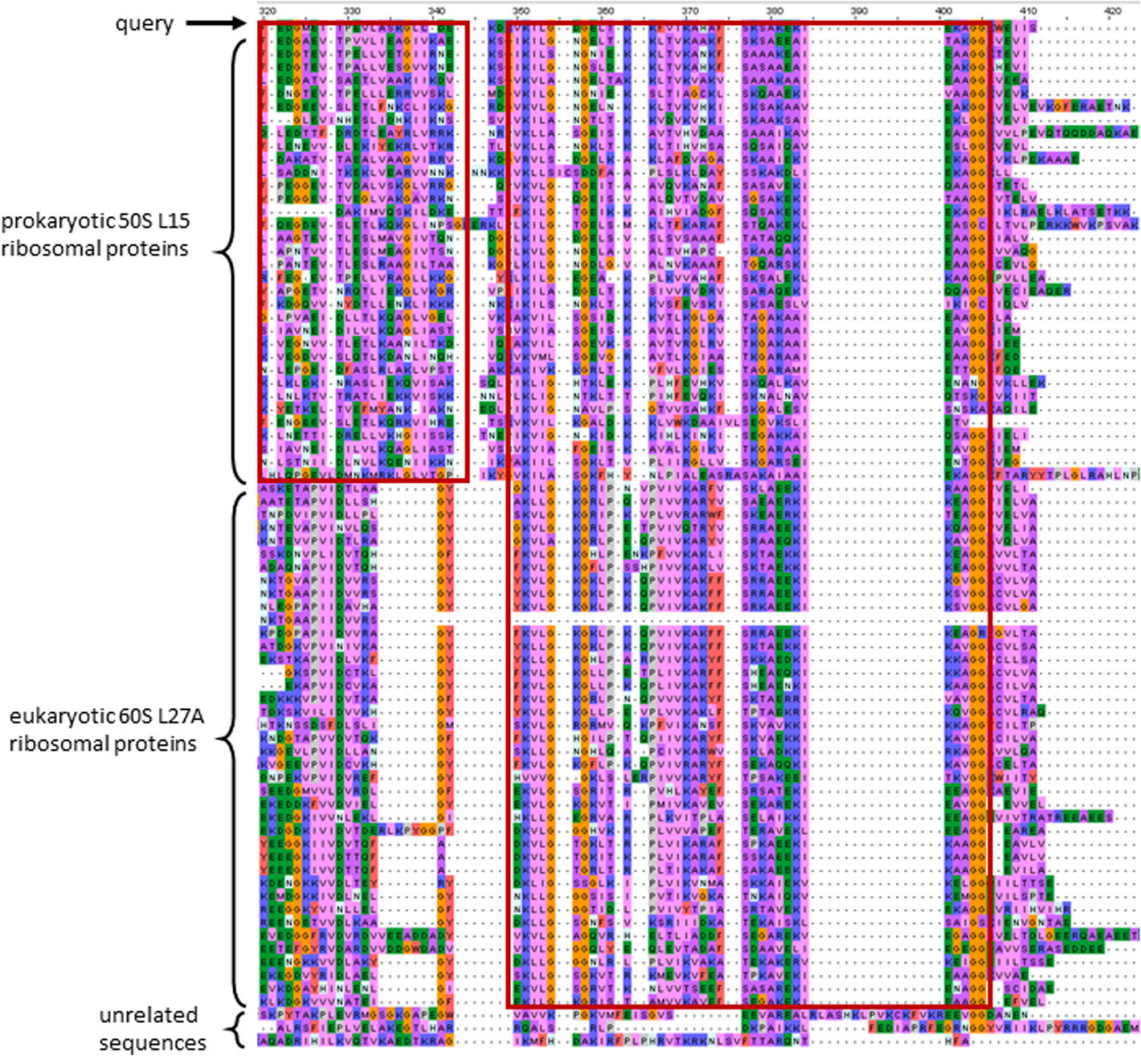
Part of an example alignment from the BAliBASE benchmark suite, aligned using MAFFT. The query sequence is from the bacteria *Aquifex aeolicus* (Uniprot:O67561) and the alignment includes both related and unrelated sequences. Conserved regions detected by LEON-BIS are outlined in red

An example of a distant sequence relationship detected by LEON-BIS, which was not identified by the original LEON algorithm, is shown in Fig. 2. The alignment (BBA0010 in the BAliBASE benchmark) contains two subfamilies, corresponding to prokaryotic 50S L15 ribosomal proteins and eukaryotic 60S L27A ribosomal proteins. The selected query sequence is from the bacteria *Aquifex aeolicus* (Uniprot:O67561)*,* and LEON-BIS successfully identified the C-terminal region that is conserved between this prokaryotic protein and the higher eukaryotic ribosomal 60S L27A (for example, the human sequence Uniprot:P46776). The predicted region is confirmed by the presence of the Prosite [27] pattern PS00475 in both prokaryotic and eukaryotic sequences.

### Detection of conserved regions and comparison with PFAM domains

**Fig. 3:**
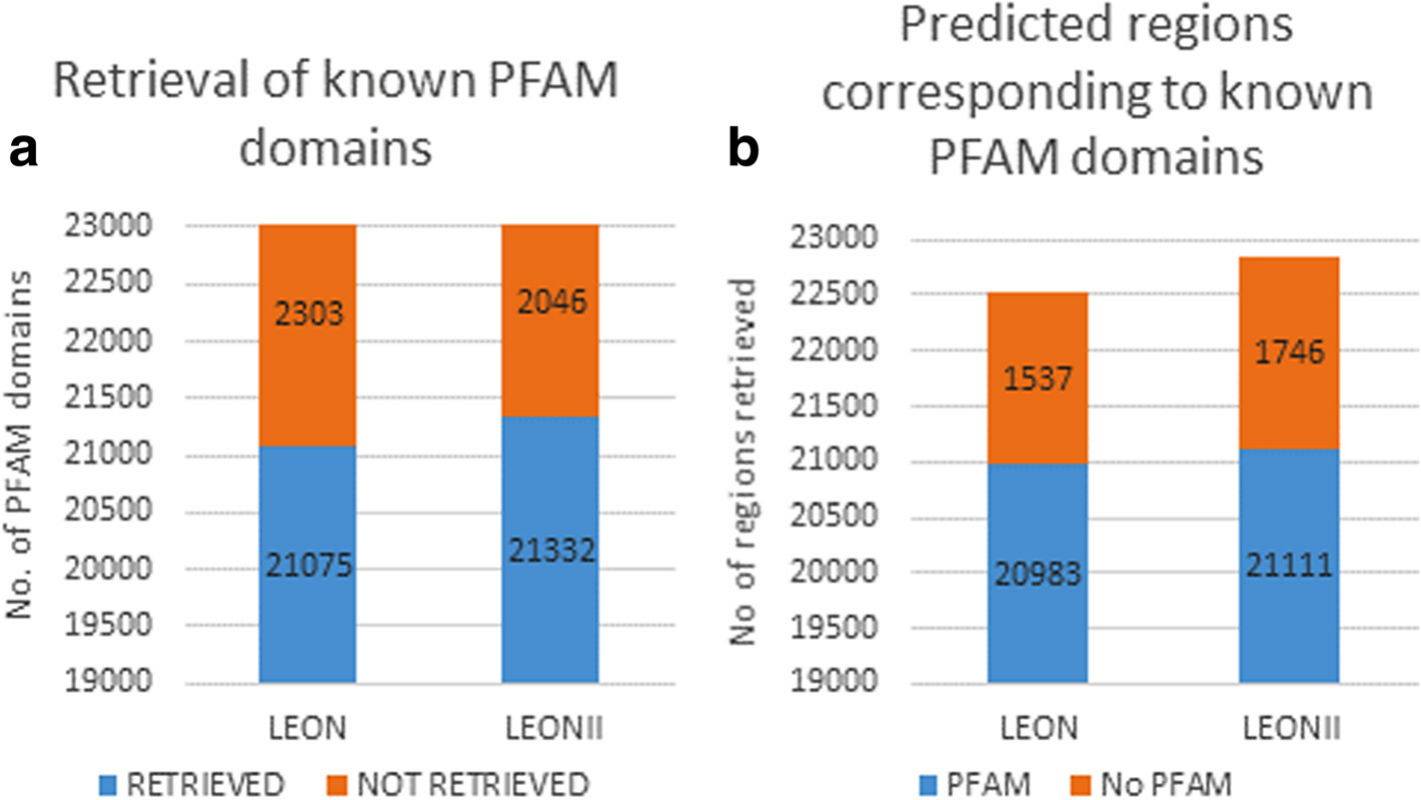
**a** Number of known domains from the PFAM protein family database successfully retrieved by the different methods tested. **b** Number of regions predicted by the different methods that overlap with known PFAM domains

To further evaluate the quality of the conserved regions predicted by LEON-BIS, we extracted the known structural or functional domains from the PFAM database [24] for all the sequence sets used in the previous evaluation. We then compared the related regions identified by LEON-BIS and by LEON with these PFAM domains, as shown in Fig. 3.

The precision (true positives/(true positives + false positives)) and recall (true positives/(true positives + false negatives)) of the two algorithms were then calculated (Table 2). Precision is defined as the fraction of retrieved instances that are relevant, i.e. the fraction of predicted regions that overlap with PFAM domains with respect to the total number of regions predicted by the method. Recall is defined as the fraction of relevant instances that are retrieved, i.e. the fraction of PFAM domains that were retrieved by the algorithms. These two measures were then combined as their harmonic mean to yield an aggregate F-measure = 2* (Precision * Recall)/(Precision + Recall), assessing the overall accuracy of the methods.

**Table 2:** Precision and recall statistics for the identification of known PFAM domains by LEON and LEON-BIS

	LEON	LEON-BIS
Recall	0.91	0.92
Precision	0.93	0.92
F-measure	0.92	0.92

**Fig. 4:**
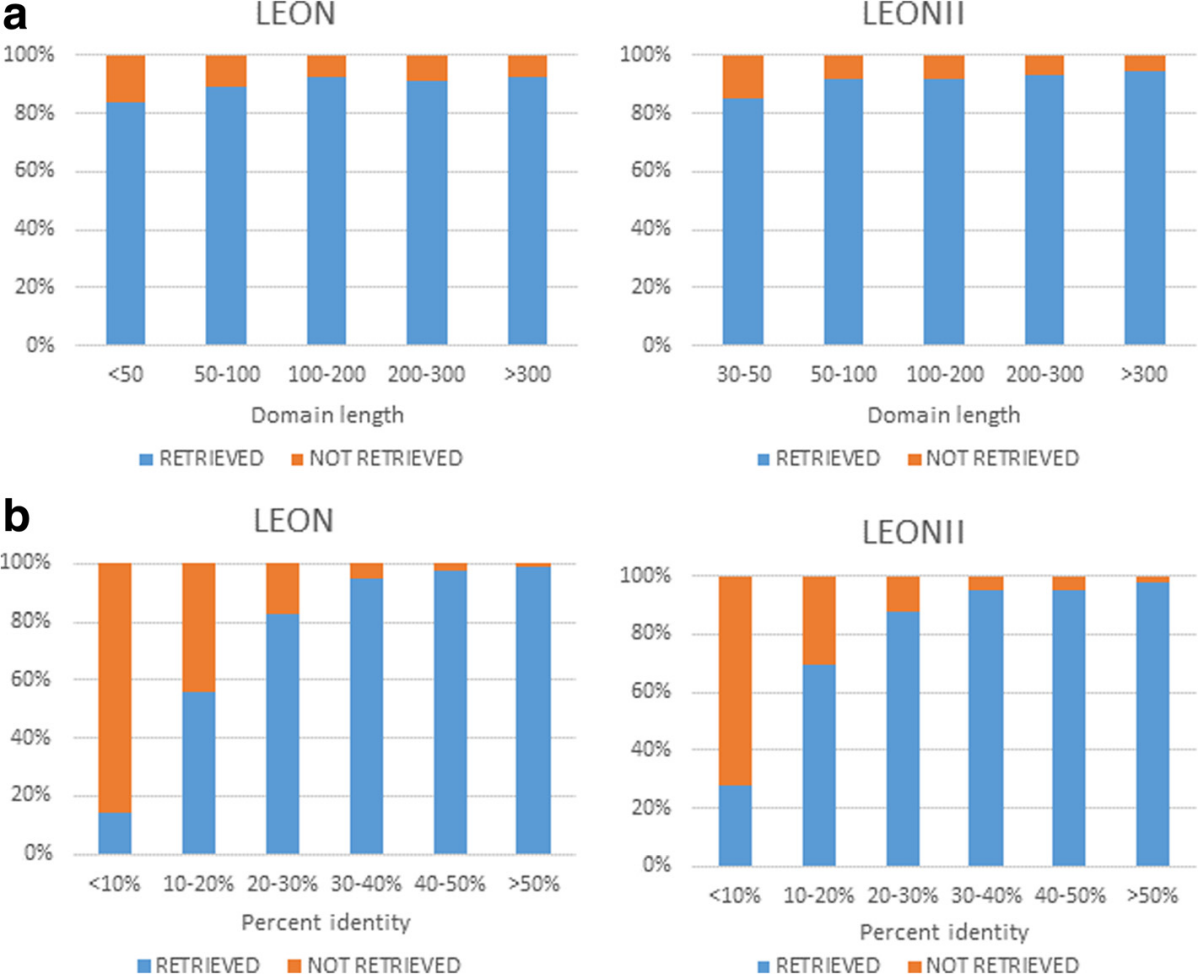
Retrieval of PFAM domains depending on **a**) domain length and **b**) percent sequence identity

Overall the precision and recall statistics are similar for LEON and LEON-BIS, however, we observed that LEON failed to retrieve 2303 PFAM domains (out of a total of 22,857), while LEON-BIS only missed 2046 domains. To further characterize their relative performances, we investigated the ability of the two methods to retrieve PFAM domains with different levels of sequence conservation and different lengths. The results are shown in Fig. 4). A small effect of sequence length was observed for both LEON and LEON-BIS with their recall power decreasing for shorter domains, especially for domains with less than 50 amino acids. As might be expected, the effect of sequence conservation is stronger, with a significant loss of recall power for both LEON and LEON-BIS for domains sharing less than 20 % residue identity. In these cases, LEON-BIS is more successful in identifying very divergent sequences, retrieving 16 out of 57 domains with less than 10 % identity, compared to 8 out of 57 for LEON.

**Fig. 5:**
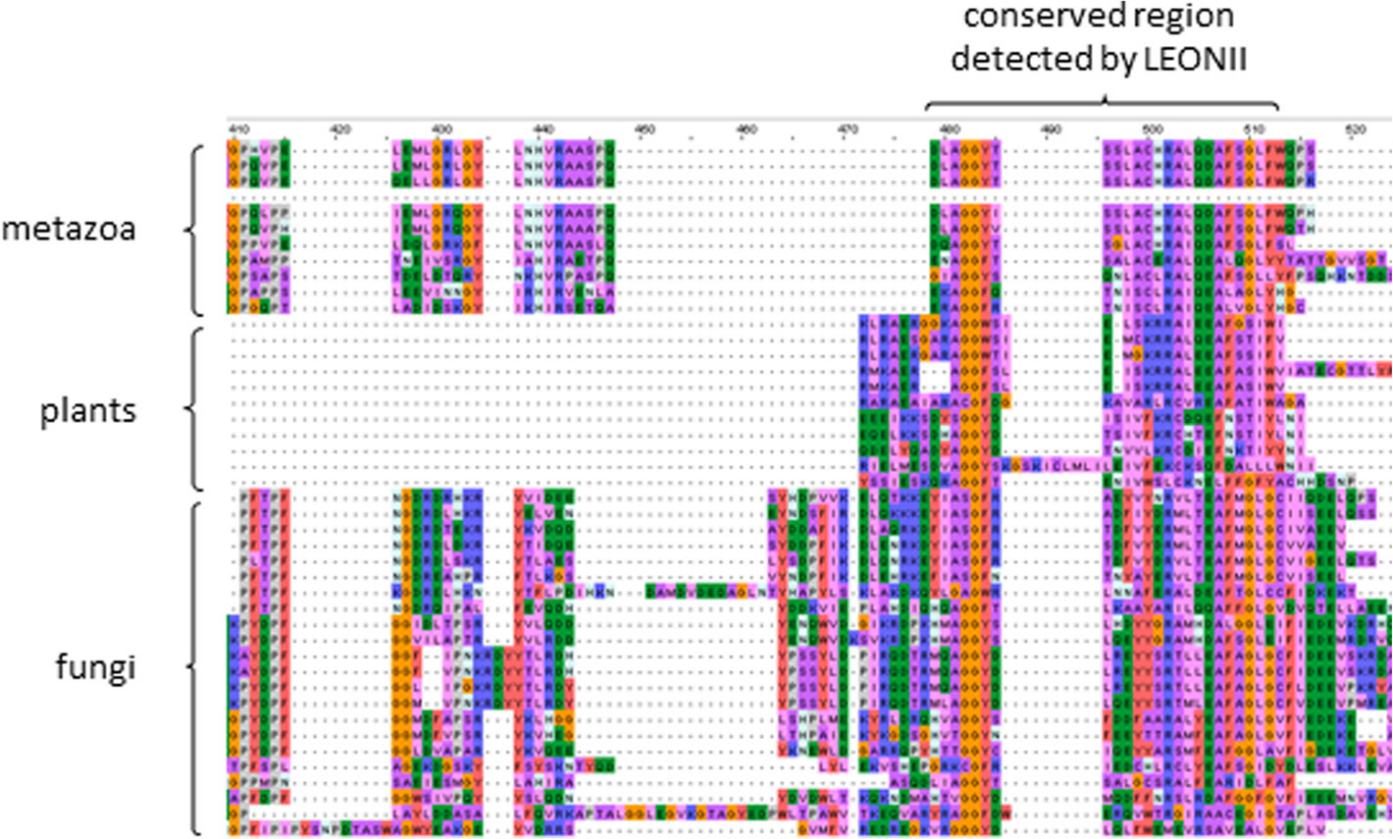
Part of the alignment constructed by MAFFT of Cdk-activating kinase assembly factor MAT1/Tfb3 sequences, showing the conserved C-terminal region

The precision of the LEON-BIS predictions in comparison to known PFAM family domains is 0.92, i.e. of the 22,857 conserved regions predicted by LEON-BIS, 1746 regions were not supported by a PFAM domain. An example of such an uncharacterized region is shown in Fig. 5. The multiple alignment (BBA0123 in the BAliBASE benchmark) contains CDK-activating kinase (CAK) assembly factor MAT1/Tfb3 sequences from metazoan, plants and fungi. MAT1/Tfb3 is a component of the general transcription and DNA repair factor IIH (TFIIH), and contains two conserved domains from the PFAM database, including the C3HC4 RING finger domain in the N-terminal region and the MAT1 domain, corresponding to a central coiled-coil domain with a long helical fibrinogen-like structure. Although the C-terminal region (positions 250–309 in the human sequence, Uniprot:P51948) does not have any PFAM annotation, LEON-BIS identified a core block that is conserved in all the metazoan, plant and fungi sequences. This block corresponds to part of the hydrophobic C-terminal domain (amino acids 229–309), identified in structural studies [28] and shown to be required to assemble and activate CAK. In yeast, truncation of the 22 C-terminal amino acids of Tfb3/Rig2 (MAT1 counterpart) was shown to be lethal [29]. Furthermore, in myeloid leukemia cells, retinoic acid (RA)-induced MAT1 fragmentation at amino acid 229 suppresses CAK phosphorylation and leads to cell cycle arrest, suggesting a novel therapeutic potential of the C-terminal protein fragment against different subtypes of myeloid leukemia [30].

The above example clearly demonstrates the value of LEON-BIS for the detection of conserved blocks or regions that may represent important new structural or functional features.

## Discussion

The determination of homology is a crucial problem for a wide range of homology-based applications, and poses particular problems in automatic, high-throughput genome analysis and annotation projects. A number of methods exist that estimate homology based on a multiple sequence alignment, but these methods generally look for features shared amongst all or most of the sequences. Alternatively, methods such as OD-seq have been developed to identify outlier sequences and remove them completely from the alignment. Nevertheless, today’s complex alignments require a more precise definition of conserved sequence segments.

Here, we have updated our original method that measured the evolutionary conservation of a set of related sequences using a frequentist approach, based on the observed amino acids in multiple alignment columns. Nevertheless, there are a number of shortcomings in estimating scores for amino acids based only on their observed frequencies, especially when the number of observed sequences is small. First, unseen amino acids are assigned scores based on an amino acid substitution matrix that is generally chosen arbitrarily. Second, an arbitrary threshold for profile scores must be defined to distinguish related from unrelated or erroneous sequences. Therefore, the profile scoring scheme, originally developed in the context of the ClustalW multiple alignment program [18], has been replaced by a Bayesian statistical framework. This allows the definition of conservation based on background knowledge (amino acid frequencies extracted from alignments and represented by Dirichlet mixture models), combined with observed amino acids in alignment columns. The Bayesian statistics are more robust than the original profiles based on observed amino acid frequencies only, as shown in the tests performed here. The Bayesian framework also means that no parameters need to be fixed by the user. The only parameters in LEON-BIS concern the chaining of blocks to form a region, i.e. the maximum distance between blocks and the minimum length of a region. In these tests, we used the same parameters as LEON for comparison purposes. Modifying these parameters would allow the user to increase either the recall or the precision, as required.

The LEON-BIS method incorporates the SIBIS algorithm, which also uses a Bayesian framework, in order to detect inconsistent or badly predicted sequences. This is an important issue when analyzing eukaryotic genome data, since recent analyses have shown that the complete exon/intron structure is correctly predicted for only about 50-60 % of genes [31]. The situation is further complicated by widespread alternative splicing events, which affect more than 92–94 % of multi-exon human genes [32]. In the presence of these inconsistent or erroneous sequences, the assumptions about amino acid distributions in the Dirichlet mixture models may not be valid. If not addressed, the estimates of amino acid probabilities would be biased, and the true unrelated sequences may not be detected as a result. By delineating the consistent sequence segments from the badly predicted sections, we can avoid excluding too many sequences. The incorporation of SIBIS also means that LEON-BIS is more robust to local misalignments. In the experiments performed here, we used multiple alignments constructed automatically with the MAFFT algorithm, rather than the high quality, manually refined reference alignments, to make the tests more realistic.

In a first large-scale evaluation, the ability of LEON-BIS to distinguish between related and unrelated sequences was compared to existing methods, including the original LEON method and a recent algorithm, OD-seq, for the detection of outlier sequences in multiple alignments. The sensitivity and specificity of LEON-BIS were shown to be slightly higher than LEON. OD-seq had very high sensitivity and detected most of the related sequences, but the specificity was low, meaning that a large number of unrelated sequences were retained in the alignments. It should be noted that OD-seq is designed specifically for very large alignments containing thousands of sequences and it therefore represents a complementary approach to the method developed here. Then, in a second experiment, the regions predicted by LEON-BIS were compared to known domains from the PFAM database and both the precision and recall of LEON-BIS were shown to be about 92 %. Compared to LEON, a significant difference was observed in the prediction of functional domains sharing low percent identity (<30 %). Furthermore, additional conserved regions were also identified in the alignments that were not covered by the existing PFAM annotations. The LEON-BIS homology predictions in combination with known structural/functional information, should therefore provide a powerful tool for the characterisation of new or unknown proteins.

## Conclusions

LEON-BIS is a fully automatic method that reliably detects conserved regions in multiple sequence alignments. It can be applied to a wide variety of alignments, including difficult cases such as distantly related sequences, multi-domain sequences, or transmembrane sequences. Incorporating LEON-BIS should therefore improve downstream applications, including phylogenetic studies (although most current methods for phylogenetic tree reconstruction cannot take into account uncertainty within alignment columns) and comparative modeling, as well as detection of sub-family specific regions representing potential specificity-determining motifs. In the future, the reliable blocks detected by LEON-BIS could provide the basis for a multi-level comparative genomics strategy, with homology analysis ranging from complete proteins to the core block level.
